# Collimation and cropping in diagnostic radiography: How concerned are we?

**DOI:** 10.1002/jmrs.648

**Published:** 2023-01-06

**Authors:** Christopher Hayre, Chandra Makanjee, Shantel Lewis

**Affiliations:** ^1^ Department of Health and Care Professions University of Exeter Exeter UK; ^2^ Department of Medical Radiation Sciences University of Canberra Canberra ACT Australia; ^3^ Department of Medical Imaging and Radiation Sciences University of Johannesburg Johannesburg South Africa

## Abstract

This editorial examines current collimation and cropping practices in general radiography. We critically reflect on whether we are concerned by the practice of collimation creep amongst radiographers. Discussions around policy, evidence‐based practice and potential hypocrisies are outlined in this editorial piece.

## Introduction

In this editorial, we examine the practice of collimation in general radiography. This examination has resurfaced in response to the ongoing identification and suboptimum use of collimation in practice. This suboptimum application has been exaggerated in response of our ability to ‘crop’ digital radiographs post‐exposure. As with digital photographs, digital X‐ray images can be ‘cropped’, naturally masking the primary area of X‐ray exposure on an image receptor. This is important because ionising radiation should be kept to a minimum to patients. We begin this editorial by asking whether we should be concerned with cropping, whereby radiographers are focused on presenting ‘the textbook image’ prior to being sent for image interpretation. In our editorial, the authors reflect on the original ethnographic work by Hayre et al.,^1^ identifying this phenomenon in 2019, empirically. Key to our discussion is the identification and ongoing disconnect between evidence and practice, coupled with the challenge of implementing evidence into practice. We will not only question potential hypocrisies in this editorial but offer a critical perspective concerning why radiographers’ may not be interested in resolving collimation creep.

## Collimation and Cropping: Should we be Concerned?

General radiographic examinations continue to remain frequently performed worldwide. Central to the practice of image acquisition is correct exposure selection, source to image distance, focal spot size and collimation. Collimation, the practice of shaping the primary field of ionising radiation, has been reported to vary amongst radiographers following the advent of digital radiography.^1^ It is generally accepted that collimation should be strictly applied, not only limiting dose area product, but improving image quality.^2^ This remains critical, in response to legislation advocating that radiological exposure, however small, is kept as low as reasonably achievable (ALARA). Currently, software packages akin to digital radiography technology allows radiographers to ‘crop’ images post‐exposure. This not only hides the initially exposed area of ionising radiation by a radiographer, on the receptor, but also masks possible overirradiation of the patient. In a previous paper, the first author attributed the use of ‘cropping’ by seeking aesthetic radiographic qualities, vis‐à‐vis, ‘a textbook image’, allowing images to appear ‘neat’ and/or ‘tidy’.^1^ This research affirmed ‘collimation creep’, whereby radiographers habitually increase the field of view with knowledge it can be corrected, post‐exposure. At the time of writing this paper it was suggested that radiographers seek to ideologically disassociate themselves from their radiographs [artwork] and instead critically reflect on inherent radiation principles and processes, coupled with obvious hinderances associated with improper collimation, as identified above. Looking back on its publication, we have been asked to critically reflect, now, leading to questions surrounding mitigation and learning experiences.

A recent study by Ball et al.,^3^ published in 2022 provides an insight into radiographic practice in a trauma centre. The study found that patients were being overexposed to radiation due to inadequate collimation of the primary beam with patients exposed to anatomy not under examination by clinicians. When comparing (and reflecting upon) these findings with those published in 2019, two features stand out. First, it reaffirms the disconnect between ‘evidence’ and ‘practice’ in radiography, suggesting that radiography is still struggling to implement empirical evidence into everyday practice, a view commonly referred as ‘implementation science’.^4^ It is possible that evidence fails to transcend into either local, national, or transnational hospital environments. At worst, it may be ignored. Second, it is possible that ‘collimation creep’ may not be receiving the attention of radiographers, educators, academics, or policymakers. For instance, a search of ‘collimation creep’ on professional body websites in the United Kingdom and Australia returns no results. It is, perhaps, unsurprising that the practice of ‘collimation creep’ remains in general radiography now and in future years, in response to little guidance or policy from professional bodies or educational institutions.

In response to the question posed: ‘collimation and cropping: should we be concerned?’ will naturally depend on whether we, as practitioners, departments, institutions and professional bodies, are ourselves concerned? Our view, as radiographers and educators writing this editorial, is that we should be recognising the suboptimum effects of poor collimation in practice whilst concerned with little policy guidance. In our view, the notion of ‘collimation creep’ must remain a topical discussion amongst colleagues, as well as other emerging practices that have the potential to increase risk to patients. Without recognition, debate and policymaking we may look back with regret, allowing collimation creep to become culturally accepted worldwide. Here, we could find ourselves grappling with some level of radiographic hypocrisy if empirical evidence is read and shared but simply fails to guide or facilitate patient outcomes.

In this editorial we not only appreciate the complexities of implementing evidence‐based radiography, but feel that some ongoing reflection, or requirement, amongst vendors would offer some shared responsibility by innovating strategies to limit or prevent cropping in the first instance. For example, this may manifest with standardising digital radiography software, whereby any action of cropping on a radiography console is readily identified on a picture archiving communication system. This, coupled with acknowledgement and guidance from professional bodies worldwide would identify increases to collimation. This feature would not only recognise suboptimum collimation, but also assist radiographers and departments alike with ways of learning and importantly improving collimation now and in future years. The bigger picture, here, seeks to improve (un)intentional behaviours amongst radiographers, which may at present be culturally accepted with increases to ionising radiation to patients. Here, the need for more qualitative and quantitative data is needed, but this is coupled with technological innovation and implementation to critical examine sensitive topics and importantly resolve or limit its practice.

## Why is Collimation not Practiced Optimally?

Both historically and contemporarily, general radiography continues to offer personalised imaging to patients.^4^ The authors in this editorial, amongst others, recognise the value of performing general imaging examinations optimally. Whilst this remains critical for healthcare delivery, we seek to question why, then, improper collimation continues in the clinical environment. First, we argue whether general imaging is merely viewed as a ‘steppingstone modality’.^5^ Little evidence suggests or hints to such claim; however, is it plausible that general radiography lacks professional standing within our community, when compared to say emergent, or established modalities considered ‘specialist roles’, such as computed tomography (CT), magnetic resonance imaging (MRI), ultrasound and/or mammography? For instance, MRI, ultrasonography and mammography are typically associated with enhanced renumeration and professional rank in the United Kingdom and Australia, alike.

Could a lack of proper collimation be practiced because general imaging is considered entry level, thus less significant, in terms of professional accountability or skill? Perhaps we, as a profession, should be asking what ‘good collimation’ is? In the last decade, in the United Kingdom, we have observed the rise of reporting practice amongst radiographers, vis‐à‐vis advanced or consultant practice, mimicking the role of radiologists. On the one hand, we recognise the overall positivity towards reporting practice and perhaps remains at the forefront of early career radiographers. Yet, on the other hand, could this progression and natural desire be silently hindering, or degrading  core radiographic skills? More importantly, are we in danger of discarding foundational practices in return for more favourable vocational practices, albeit for remuneration, image interpretation, or cross‐sectional roles, respectively, which have historically sat outside a radiographer's scope of practice? This shift towards imaging interpretation has been warned amongst others whereby more reflexivity is needed in viewing radiographs,^6^ with the first author, later, suggesting potential deskilling.^7^ Reflexivity is important because it can enable individuals to critically examine actions or behaviours pertinent to image acquisition. This raises a broader question as to whether obvious increases to dose area product, in response to overcollimation, are deemed ‘risk‐free’ or ‘risk‐irrelevant’ with perhaps a perceptual paradigm shift away from the linear non‐threshold model. In this argument, upon graduation, radiographers may seek to ‘move on’ from general radiography and into some other specialised role, when possible. Whether driven by renumeration, personal interest, or career goals, the continuation and acceptance of improper collimation suggests that some radiographers may be less motivated and/or encouraged to apply foundational radiographic principles, such as sound collimation in the first instance and, importantly, keep doses as low as reasonably practicable.

This critical examination into collimation and collimation creep is important for two reasons. First, looking back on the first authors research in 2019, which questioned the phenomena ‘collimation creep’, we are clearly observing this practice translationally, 4 years after its publication. Importantly, we should ask why ‘good’ collimation is still not practiced appropriately and perhaps begin departmental, institutional or policy driven approaches to reflect and limit ionising radiation to patients. Are we as a profession silently responding to an alternate radiobiological paradigm, avoiding practice issues, or perhaps evolving away from observing general radiography as a modality worthy of such debate because doses are too small for concern? Whilst we suspect this is not widely accepted amongst managers or practitioners alike, we should start to ask questions about general imaging that remain unchallenged, whether due to advancing technology, or in response to radiographic practice that is readily hidden and rectified using post‐processing software.

## Conclusion

In this editorial we sought to question the practice of collimation in general radiography and identify current and future challenges. We began by asking whether we should be concerned by reflecting on a recent audit identifying improper collimation in Australia, whilst looking back on the first authors original ethnographic work conducted in the United Kingdom, identifying this practice. Some important discussions arose, notably the implementation of evidence‐base practice remaining a significant challenge in medical imaging. Further, we recognise that a combination of protocol development, coupled with innovation in software and policy making is needed to confront this challenge and prevent its occurrence. If not, we may find ourselves battling with some level of professional hypocrisy if we do little, or at worse, nothing. Lastly, we offer a critique of general radiographic practice, whereby our reluctance to limit improper collimation is grounded by it being considered a ‘steppingstone modality'. In addition, should we start to question our everyday professional practice as we continue to shift in becoming image interpretation experts, and possibly leave behind image acquisition expertise.

## Conflict of Interest

The authors declare no conflict of interest.
